# Study on the Main Components Interaction from *Flos Lonicerae* and *Fructus Forsythiae* and Their Dissolution In Vitro and Intestinal Absorption in Rats

**DOI:** 10.1371/journal.pone.0109619

**Published:** 2014-10-02

**Authors:** Wei Zhou, Xiaobin Tan, Jinjun Shan, Shouchuan Wang, Ailing Yin, Baochang Cai, Liuqing Di

**Affiliations:** 1 College of Pharmacy, Nan’jing University of Chinese Medicine, Nan’jing, People’s Republic of China; 2 Jiang’su Engineering Research Center for Efficient Delivery System of TCM, Nan’jing, People’s Republic of China; 3 Nan’jing Engineering Research Center for Industrialization of Chinese Medicine Pellets, Nan’jing, People’s Republic of China; 4 Laboratory of New Drug Delivery System of Chinese Meteria Medica, Jiang’su Provinical Academy of Chinese Medicine, Nan’jing, People’s Republic of China; 5 Jiangsu Key Laboratory of Pediatric Respiratory Disease, Institute of Paediatrics, Nan’jing University of Chinese Medicine, Nan’jing, People’s Republic of China; Macau University of Science and Technology, Macao

## Abstract

The *Flos Lonicerae-Fructus Forsythiae* herb couple is the basic components of Chinese herbal preparations (Shuang-Huang-Lian tablet, Yin-Qiao-Jie-Du tablet and Fufang Qin-Lan oral liquid), and its pharmacological effects were significantly higher than that in *Flos Lonicerae* or *Fructus Forsythiae*, but the reasons remained unknown. In the present study, pattern recognition analysis (hierarchical cluster analysis (HCA) and principal component analysis (PCA)) combined with UHPLC-ESI/LTQ-Orbitrap MS system were performed to study the chemical constitution difference between co-decoction and mixed decoction in the term of chemistry. Besides, the pharmacokinetics *in vivo* and intestinal absorption *in vitro* combined with pattern recognition analysis were used to reveal the discrepancy between herb couple and single herbs in the view of biology. The observation from the chemical view *in vitro* showed that there was significant difference in quantity between co-decoction and mixed decoction by HCA, and the exposure level of isoforsythoside and 3, 5-dicaffeoylquinic acid in co-decoction, higher than that in mixed decoction, directly resulted in the discrepancy between co-decoction and mixed decoction using both PCA and HCA. The observation from the pharmacokinetics displayed that the exposure level *in vivo* of neochlorogenic acid, 3, 4-dicaffeoylquinic acid, isoforsythoside and forsythoside A, higher than that in single herbs, was the main factor contributing to the difference by both PCA and HCA, interestingly consistent with the results obtained from Caco-2 cells *in vitro*, which indicated that it was because of intestinal absorption improvement of neochlorogenic acid, 3, 4-dicaffeoylquinic acid, isoforsythoside and forsythoside A that resulted in a better efficacy of herb couple than that of single herbs from the perspective of biology. The results above illustrated that caffeic acid derivatives in *Flos Lonicerae*-*Fructus Forsythiae* herb couple could be considered as chemical markers for quality control of its preparations.

## Introduction

Herbs used together in couples (Yaodui in Chinese) are the basic composition units of Chinese herbal formulas and have special clinical significance in Traditional Chinese Medicine (TCM). The herb couples are much simpler than other complicated formulas yet retain the basic therapeutic features. It is important to elucidate the compatibility foundation of TCM formulas by investigating the law and effective substance of herb couples, which are the major areas of research supported by Chinese government [1]. There are magnitudes of methods to study the compatibility rule and principles of herb couples to some extent including literature research [2], extraction and separation [3], pharmacological effects [4]–[6], pharmacokinetics *in vivo* of active components [7] and serum pharmaco-chemistry methods [8], *etc*. However, up to now, few studies were performed to explain the reasonability of herb couples based on the systemic biopharmaceutics both *in vivo* and *in vitro*.

*Flos Lonicerae* (FLJ) possesses wide pharmacological actions, such as antibacteria, anti-inflammation, antivirus, antiendotoxin, blood fat reduction, *etc* [9], and *Fructus Forsythiae* (FF) has antibacterial, antiviral, antioxidant, anti-inflammatory, anti-obesic effects, *etc* [10]. The two herbs are the basic components of Chinese herbal preparations such as Shuang-Huang-Lian oral liquid, Yin-Qiao-Jie-Du tablet, Qin-Re-Jie-Du oral liquid and Fufang Qin-Lan oral liquid, which are extensively used in clinical practice [11], and it was shown by Lin (2008) [12] that the pharmacological effects such as anti-inflammatory and antipyretic effects in FLJ-FF herb couples were significantly stronger than that in FLJ or FF, but the reasons were unknown. Thus, it was presumed that the dissolution *in vitro* of main ingredients was significantly higher than that of FLJ and FF or the intestinal absorptions of active components were improved combined with FLJ or FF.

In short, the current study aims to demonstrate the optimal efficacy of FLJ-FF herb couple based on thoughts we provided above analyzed by both hierarchical cluster analysis (HCA) and principal component analysis (PCA) for the further development of herb couples preparations. The specific objectives of the current study include: (1) To study the difference between co-decoction and mixed decoction based on the dissolution *in vitro*. (2) To research the discrepancy between herb couple and single herbs based on the pharmacokinetics *in vivo.* (3) To illustrate the difference via *in vitro* Caco-2 cells between herb couple and single herbs.

## Materials and Methods

### Ethics statement

All procedures had the approval of the Animal Ethics Committee of the Nanjing University of Chinese Medicine.

### Reagents and chemicals

FLJ (bud of *Lonicera japonica* Thunb.) and FF (fruit of *Forsythia suspense*) were purchased from Yi-Feng drug store (Nanjing, China) and authenticated by Prof. Wu (Department of Pharmacognosy, Nanjing University of Chinese Medicine). All voucher specimens were deposited in our laboratory for future reference. Chlorogenic acid, luteoloside, pillyrin, forsythoside A and tinidazole (using as internal standard, IS) were purchased from National Institute for the Control of Pharmaceutical and Biological Products (Beijing, China). Neochlorogenic acid, cryptochlorogenic acid, 3, 4-dicaffeoylquinic acid, 3, 5-dicaffeoylquinic acid and forsythoside B (98% pure) were purchased from Sichuan Weikeqi Bio-tech Co., Ltd. (Sichuan, China). Isoforsythoside, caffeic acid, quinic acid, genistein, luteolin, quercetin, arctigenin, genistin, astragalin, hyperoside, isoquercitrin, pinoresinol-β-D-glucoside, arctiin, rutin, dipsacoside B and loganin (98% pure) were purchased from Chengdu Herbpurify Co., Ltd. (Sichuan, China). Heparin sodium injection was purchased from Changzhou Qianhong Bio-pharma Co., Ltd. (Changzhou, China). Methanol and acetonitrile (HPLC grade) were purchased from Merck (Merck, Germany), and water was purified by a Milli-Q water purification system (Millipore, Bedford, MA, USA). All other chemicals and reagents were of analytical grade.

Trifluoroacetic acid, Lucifer yellow (LY) and DMSO were purchased from Sigma Chemical Co. (St. Louis, MO). Phosphoric acid, acetic acid, formic acid, methanol and acetonitrile (HPLC grade) were purchased from Merck (Merck, Germany), and water was purified by a Milli-Q water purification system (Millipore, Bedford, MA, USA). All other chemicals and reagents were of analytical grade.

Dulbecco’s modified Eagle’s medium (DMEM), fetal bovine serum (FBS), 0.05% trypsin-EDTA, penicillin-streptomycin and non-essential amino acids were obtained from GibcoBRI, Life and Technologies, USA. Collagen type I, sodium pyruvate, MTT (3-(4, 5-dimethylthiazole-2-yl)-2, 5-diphenyl tetrazolium bromide) and trypsin_TPCK (Tosylamide Phenylethyl Chloromethyl Keton-treated Trypsin) were purchased from Sigma Chemical Co. (St. Louis, MO, USA). HBSS (Hank’s balanced salt solution) and PBS (Phosphate Buffered Saline) were purchased from Sigma Chemical Co. (St. Louis, MO, USA). Culture cell inserts for 6 well plates (CCI, 137435) were purchase from Nalge Nunc International. (Roskilde, Denmark).

The human colorectal cancer cell lines (Caco-2, HCT116) were bought from cell bank (Chinese Academy of Sciences).

Male Sprague-Dawley (SD) rats (∼250 g) were supplied by the Experimental Animal Center of Nanjing University of Chinese Medicine (Certificate No. SCXK2008-0033). The experimental procedures were in compliance with the animal ethics committee of the Nanjing University of Chinese Medicine.

### Preparation of mixed decoction and co-decoction

FLJ (30.0 g, 40 meshes) and FF powders (30.0 g, 40 meshes) were decocted with boiling water (1∶10, *w*/*v*) for 60 min by heating reflux, respectively. The two extracted solutions were filtered through 5 layer gauzes with the concentrations of 100 mg raw medicine per milliliter, respectively. The mixed decoction was prepared by mixing the two extracted solutions (1∶1, *v*/*v*).

FLJ (15.0 g, 40 meshes) combined with FF powders (15.0 g, 40 meshes) (1∶1(*w*/*w*)) as co-decoction were decocted with boiling water (1∶10, *w*/*v*) for 60 min by heating reflux. The extracted solution was filtered through 5 layer gauzes with the concentrations of 100 mg raw medicine per milliliter.

### Preparations of FLJ and FF concentrated extracts

**Figure 1:**
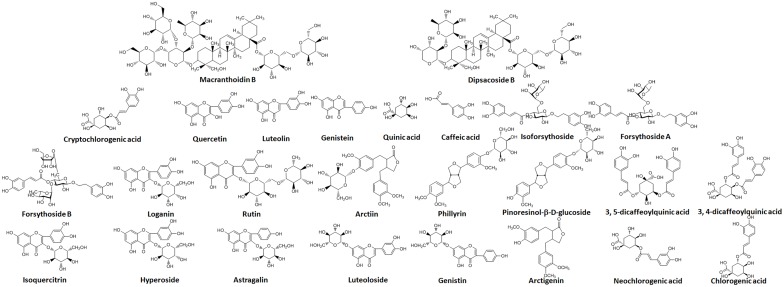
Structural formulae of analyte standards in FLJ-FF herb couple including flavones, organic acids, saponins, iridoids, phenylethanoid glycosides and lignans.

FLJ (1000 g) and FF (1000 g) were decocted twice with boiling water (1∶10, *w*/*v*) for 45 min, respectively. The two extracted solutions were filtered through 5 layer gauzes, and concentrated to a thick solution with the concentration of 2.5 g raw medicine per milliliter with 400 mL of extract used as follows, respectively [12], and the contents of caffeic acid, quinic acid, neochlorogenic acid, chlorogenic acid, cryptochlorogenic acid, 3, 5-dicaffeoylquinic acid, 3, 4-dicaffeoylquinic acid, genistin, luteoloside, astragalin, hyperoside, isoquercitrin, rutin, genistein, luteolin, quercetin, macranthoidin B, dipsacoside B and loganin (Fig. 1) in FLJ extract were 246.70, 29795, 8022.0, 24900, 7642.5, 3371.3, 5174.1, 17.313, 570.04, 43.595, 1044.0, 47.244, 789.64, 0.99522, 49.549, 20.007, 2.8651, 4.0725 and 542.44 µg/mL, respectively. And the contents of caffeic acid, quinic acid, rutin, hyperoside, isoquercitrin, quercetin, pinoresinol-β-D-glucoside, pillyrin, arctiin, arctigenin, isoforsythoside, forsythoside A and forsythoside B (Fig. 1) in FF extract were 118.16, 1092.2, 858.11, 1435.1, 4.2824, 79.045, 1958.9, 1380.1, 81.296, 17.313, 1356.1, 8036.0 and 826.97 µg/mL, respectively.

### Rat *in* *vivo* pharmacokinetics study

**Product A (extract mixed with FLJ extract and water, (1∶1,** ***v*** **/** ***v*** **)), product B (extract mixed with FF extract and water, (1∶1,** ***v*** **/** ***v*** **)) and product C (extract mixed with FLJ and FF extracts, (1∶1,** ***v*** **/** ***v*** **))** were given to oral administration to rats, respectively.

**Figure 2:**
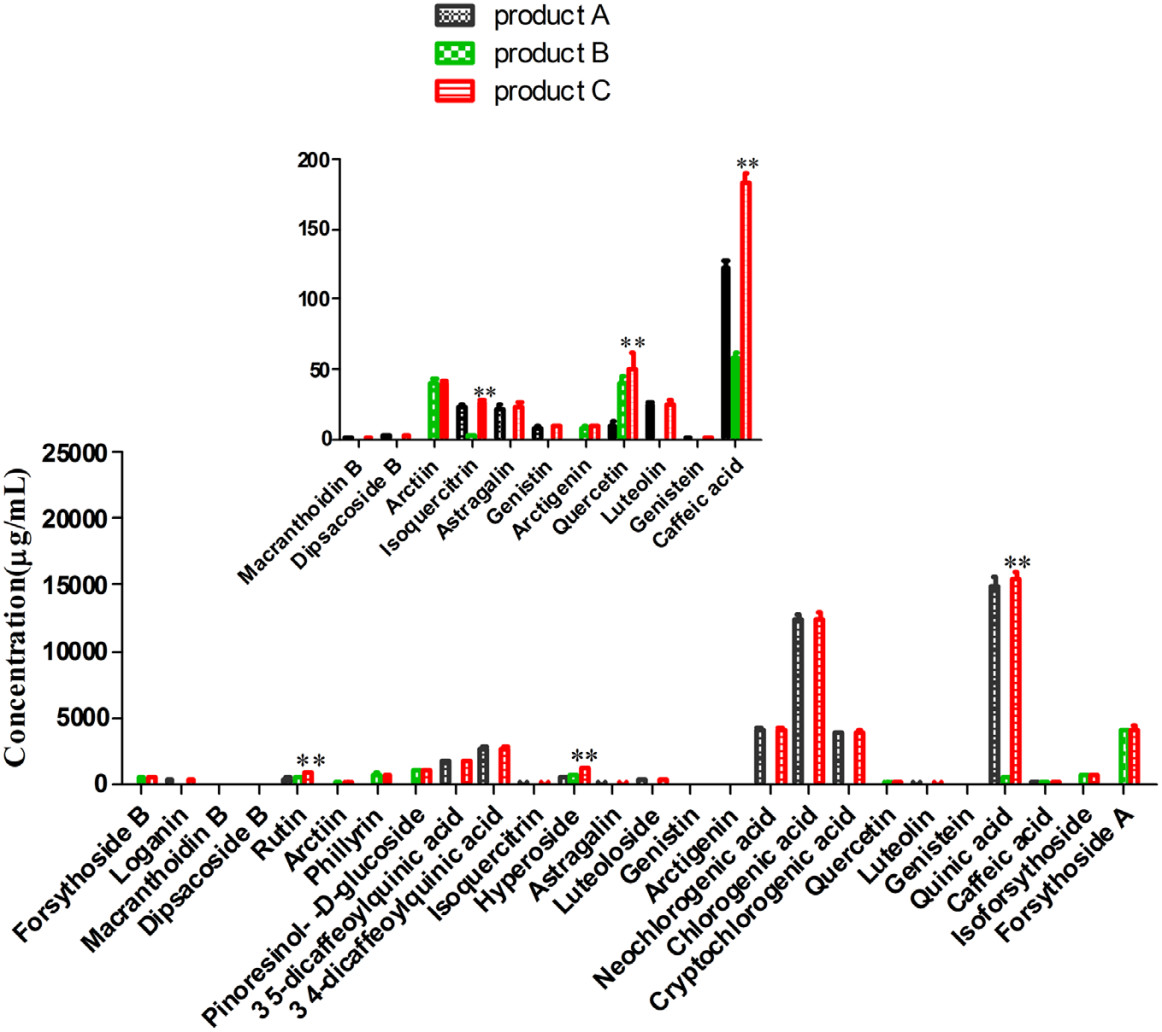
The contents of product A, B and C groups analyzed by UPLC-ESI-MS/MS. (*) *P*<0.05 and (**) *P*<0.01, compared with product C group.

In order to study the pharmacokinetics *in vivo* based on drug-drug interaction of main ingredients between FLJ and FF, oral administration to rats at the same concentration was necessary, and we found that there was no significant difference of ingredients in product A or product B except caffeic acid, quinic acid, rutin, hyperoside, isoquercitrin and quercetin in quantity (Fig. 2), compared with that in product C group.

Male SD rats (∼250 g) were kept in an environmentally controlled breeding room (temperature: 20±2°C, relative humidity: 60±5%) for 1 week. The animals were fasted for 12 h prior to drug administration. The rats were randomly divided into three groups with no less than six rats in each group to receive various administrations at a single oral dose (10 mL·kg^−1^) by gastric gavage. After dosing for 0, 5, 10, 20, 30, 40, 55, 70, 100, 160, 250, 600 and 1440 min, blood was collected from the pre-intubated catheter and put into tubes with heparin sodium injection (10 µL) at predetermined time points. Subsequently, plasma was prepared by centrifugation at 1,816×g, 4°C for 7 min, and immediately analyzed or stored at −70°C for further analysis.

### *In vitro* Caco-2 monolayer model

Caco-2 cells were cultured in high glucose DMEM with 10% fetal bovine serum, 1% nonessential amino acids. Cells were cultured in a humidified atmosphere of 5% CO_2_ at 37°C. After reaching 80% confluens, Caco-2 cells were harvested with 0.05% trypsin-EDTA solution and seeded on top of CC inserts in 6-well plates, which has a surface area of 4.2 cm^2^, at a density of 1.0×10^5^ cells/cm^2^. The protocols for cell culture in Transwell inserts were similar to those described previously [11].

Hank’s balanced salt solution (HBSS) was used as the transport buffer for the transport study in Caco-2 cells monolayer model. It was prepared by dissolving 9.5 grams of commercial available HBSS powder in 1000 mL water. The pH value of the buffer was adjusted to pH 6.0 by 85% of phosphoric acid.

MTT test was used to estimate the potential cytotoxicities of the studied product A, product B and product C toward Caco-2 cells. The Caco-2 cells were seeded onto a 96-well plate at a seeding density of 5×10^4^ cells/well in DMEM culture medium and cultured at 37°C for 24 h. Subsequently, the culture medium was replaced with 100 µL of product A, product B and product C dissolved in HBSS (pH 6.0) at different studied concentrations. Blank HBSS (pH 6.0) was employed as a negative control. Then the 96-well plate was incubated at 37°C for 24 h. Thereafter, 20 µL of 5 mg/mL MTT solution in HBSS was added to each well and the plate was incubated for another 4 h. The solutions in each well were then removed followed by dissolving the remained formazan crystals in the cells with 200 µL of DMSO. The absorbance of the mixture in the 96-well plate was then measured with a Kinetic microplate reader (Molecular Devices) at 570 nm. The cytotoxicity of each compound was calculated as the percentage of the absorbance relative to that of the negative control.

Cell culture experiments were described previously [11]. Briefly, after culture medium was aspirated, the cell monolayers were washed three times with blank HBSS. The transepithelial electrical resistance (TEER) values of cell monolayers were measured, which were more than 250 Ω×cm^2^. The monolayers were incubated with the blank HBSS for 1 h with 37°C. Thereafter the incubation medium was aspirated. Afterwards, a solution containing the compound was loaded onto the apical side. The amounts of transported compound were measured as a function of time. Donor samples (400 µL) (Apical side) and receiver samples (400 µL) (Basolateral side) were taken at different times (typically 1 h), followed by the addition of 400 µL drug donor solution to the donor side (AP) and 400 µL of blank buffer to the receiver side (BL). The samples were taken at 0, 1, 2, 3 and 4 h after incubation. At the end of the transport experiment, integrity of the monolayer was monitored by TEER value.

#### Chemical sample (mixed decoction and co-decoction) analyzed by UHPLC-ESI/LTQ-Orbitrap-MS

Mixed decoction and co-decoction were diluted five times with methanol, respectively, and then filtered through 0.22 µm membrane before injection into the UHPLC-ESI/LTQ-Orbitrap-MS system for analysis.

The UHPLC analysis was performed on the Dionex UltiMate 3000 analytical system acquired from Fisher Scientific (Thermo Fisher scientific, Waltham, MA, USA) that consisted of an autosampler equipped with a column oven, a tray compartment cooler, and a quaternary pump with a built in solvent degasser, all piloted by Xcalibur software. The chromatographic separation was achieved using Syncronis C_18_ column (100×2.1 mm, 3 µm) (Thermo Scientific, Waltham, MA, USA). The injection volume used was 5 µL. The mobile phase was composed of A (acetonitrile) and solvent B (0.05% formic acid, *v*/*v*) with a linear gradient elution: 0–3 min, 5–5% A; 3–25 min, 5–22% A; 25–30 min, 22–60%; 30–35 min, 60–95% A; 35–36 min, 95–5% A, and hold for 4 min, at a flow rate of 400 µL/min, resulting chromatographic full-width-at-half-maximum (FWHM) of 3–5 s. The column oven and tray cooler temperatures were set to 30 and 4°C, respectively.

The Dionex UltiMate 3000 UHPLC system was hyphenated with a LTQ-Orbitrap XL mass spectrometer (Thermo Scientific, Waltham, MA, USA). The system was equipped with an ESI source, operated in positive ionization mode using the following parameters: capillary temperature 350°C, capillary voltage 47 V, sheath gas flow 30 arbitrary units, auxiliary gas 8 arbitrary units, source voltage 4.5 kV and tube lens voltage 130 V. Spectra was recorded in the range of *m/z* 50–1500 Da with a resolution of 30,000. For identification purposes, two scan events were applied for the MS experiments. The first was in a full scan MS mode and the second was a data dependent scan that selected the most intense ion or specified ions in another setting from the first scan event for the acquisition of MS/MS spectra. The collision energy for collision-induced dissociation or high-collision energy dissociation mode was adjusted to 35% of maximum, and the isolation width of precursor ions was *m/z* 2.0 Da. In the qualitative study, the compounds were identified by comparison with reference compounds when available and with literature data, from retention times, MS, MS/MS and MS^n^ analysis. In the quantitative study, full scan MS mode was applied, and then extracted ion chromatograms were generated from their theoretical exact masses using a mass tolerance of 5 ppm. After integration of the peaks, their respective areas were used for the quantifications.

#### Chemical sample (product A, product B and product C) quantified by UPLC-ESI-MS/MS

Product A, product B and product C were diluted five thousand times with 10% acetonitrile/methanol (4∶1, *v*/*v*) containing 0.4% formic acid and 0.5 mM sodium formate, respectively, and then filtered through 0.22 µm membrane before injection into the UPLC-ESI-MS/MS system for analysis. The methodology validations have been studied and to be published elsewhere.

#### Biological samples (pharmacokinetics in vivo and Caco-2 cells in vitro) quantified by UPLC-ESI-MS/MS

The treatment and UPLC-ESI-MS/MS analysis for samples collected from *in vitro* and *in vivo* models, respectively have been studied and to be published elsewhere.

### Calculation

For Caco-2 monolayer model, the apparent permeability coefficient (*P*_app_) was calculated as *P*_app_ = [(dQ/dt)]/[A×C], d*Q*/d*t* (µg/S) was the flux rate, *A* was the effective surface area of the cell monolayer (4.2 cm^2^), and *C*_0_ (µg/mL) was the initial drug concentration in the donor chamber.

### Pharmacokinetic analysis

The main pharmacokinetic parameters including the peak plasma concentration (*C*_max_), the time to *C*_max_ (*T*_max_), the *AUC* from 0 to infinity (*AUC*_0–∞_), the *AUC* form 0 to time (*AUC*_0–t_), mean residence time (*MRT*), and terminal elimination half-life (*T*_1/2z_) were calculated by the non-compartmental analysis of plasma concentration *vs*. time data using the “DAS 2.1.1” software (Mathematical Pharmacology Professional Committee of China, Shanghai, China). The comparison of pharmacokinetic parameters was possessed by SPSS 20.0 (Statistical Package for the Social Science).

### Statistical analysis

Statistical significance in the *P*_app_ values and pharmacokinetic parameters were estimated by the analysis of variance (Student t-test) or one-way ANOVA. A *p* value of less than 0.05 was considered to be significantly different. All data were expressed as mean±SD.

### Pattern recognition analysis

The data for chemical difference between co-decoction and mixed decoction, and intestinal absorption both *in vitro* and *in vivo* were all visualized by applying pattern recognition methods, such as HCA and PCA, which has been extensively applied to the biomolecules analysis [13]–[16]. Both HCA and PCA were done by SPSS 20.0 software. Between-group linkage method was applied, and squared Euclidean distance was selected as measurement.

## Results

### Chemical constitution difference between co-decoction and mixed decoction by pattern recognition analysis

**Figure 3:**
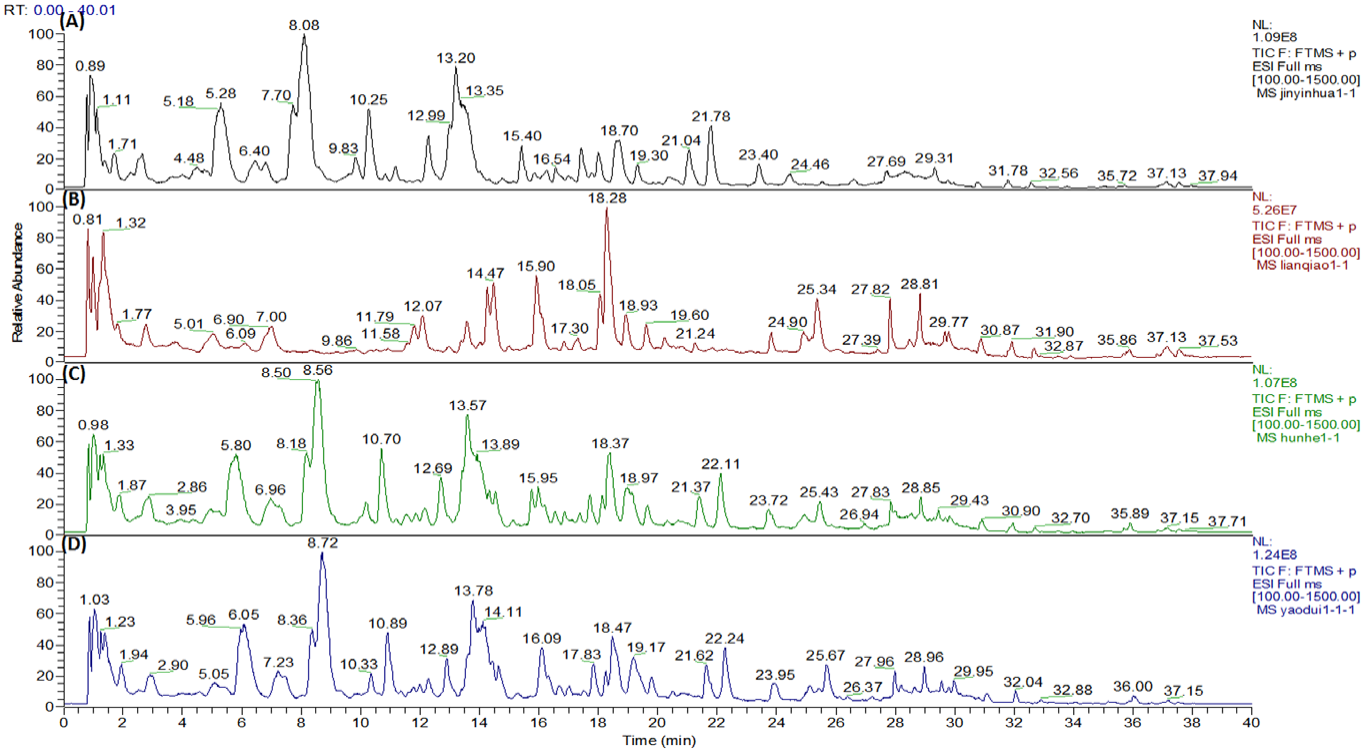
The TIC chromatographies of FLJ group (a), FF group (b), mixed decoction (c) and co-decoction (d) analyzed by UHPLC-ESI/LTQ-Orbitrap-MS.

**Figure 4:**
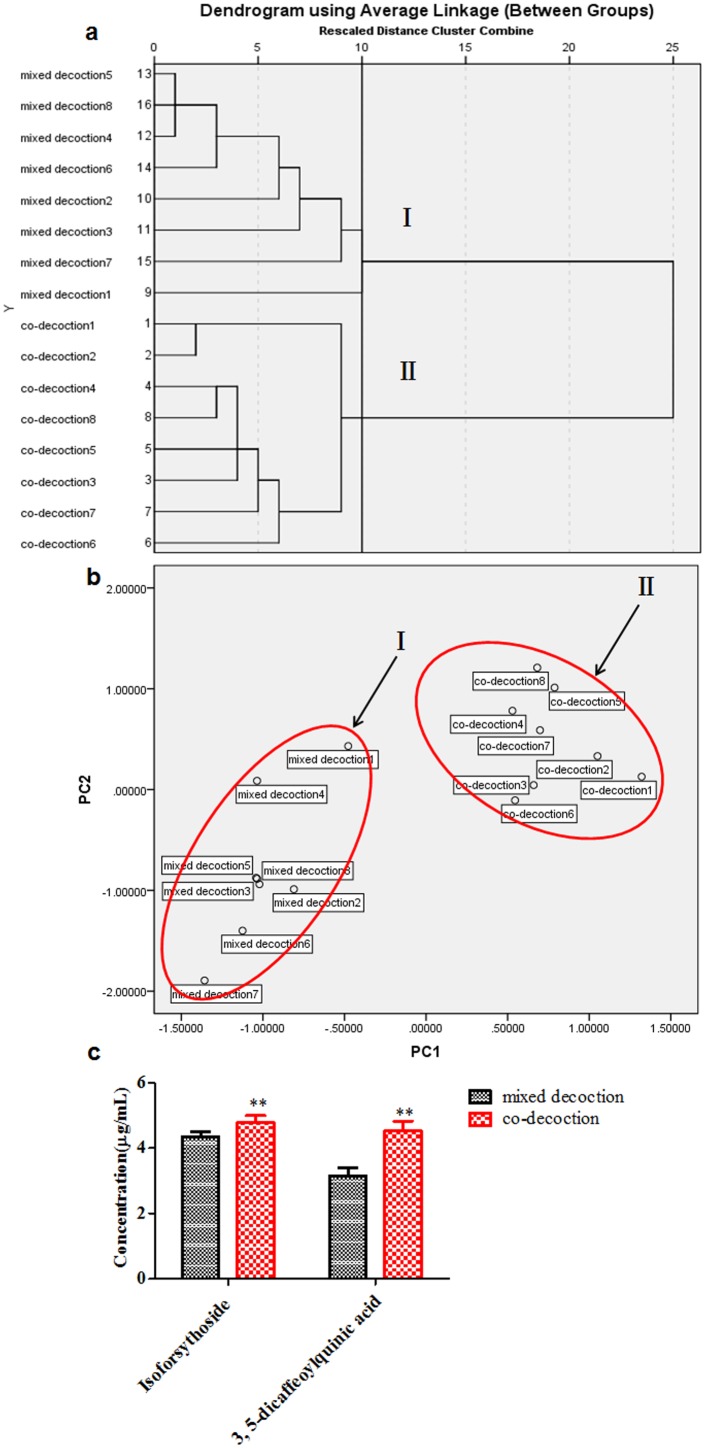
The chemical constitution differences between co-decoction and mixed decoction analyzed by both HCA and PCA. (A: HCA; B: PCA; C: the most important ingredients influencing the difference between co-decoction and mixed decoction).

As illustrated in Fig. 3A, B and Table 1, we found that 32 peaks and 31 peaks were detected in FLJ extract and FF extract in the positive ion model by UHPLC-ESI/LTQ-Orbitrap MS system, respectively and the influence of the co-decoction on the quality was little, compared with the mixed decoction (Fig. 3C, D). However, it was shown (Fig. 4a) that there was significant difference in quantity between mixed decoction and co-decoction analyzed by HCA, and we found (Fig. 4b, c) that the most possible ingredients resulted in the discrepancy between co-decoction and mixed decoction were isoforsythoside and 3, 5-dicaffeoylquinic acid analyzed by PCA combined with HCA. Besides, the contents of isoforsythoside and 3, 5-dicaffeoylquinic acid in co-decoction were significantly higher than that in mixed decoction, which indicated that isoforsythoside and 3, 5-dicaffeoylquinic acid might be the main chemical markers in herb couples.

**Table 1:** The chemical components identified from mixed decoction and co-decoction of *Flos Lonicera-Fructus Forsythiae* herb couple.

Peak no.	Components	Chemical formula	ESI^+^, *m/z*	Ret. time	Source
			MS, MS*^n^*		
1	Quinic acid	C_7_H_12_O_6_	193.07066 [M+H]^+^	1.16	FLJ, FF
2	Forsythoside D	C_20_H_28_O_13_	499.14294 [M+Na]^+^	2.32	FF
3	Neochlorogenic acid	C_16_H_18_O_9_	355.10236 [M+H]^+^, 162.97160	2.98	FLJ
4	Caffeic acid	C_9_H_8_O_4_	181.04884 [M+H]^+^	3.00	FLJ, FF
5	Forsythoside E	C_20_H_30_O_12_	485.16295 [M+Na]^+^, 185.12677	5.19	FF
6	Chlorogenic acid	C_16_H_18_O_9_	355.10236 [M+H]^+^, 162.97160	6.05	FLJ
7	Cryptochlorogenic acid	C_16_H_18_O_9_	355.10236 [M+H]^+^, 162.97160	7.23	FLJ
8	Sweroside	C_16_H_22_O_9_	359.13366 [M+H]^+^, 126.99121,179.00241	10.89	FLJ
9	Loganin	C_17_H_26_O_10_	391.15987 [M+H]^+^	13.38	FLJ
10	Centauroside	C_34_H_46_O_19_	759.27061 [M+H]^+^	14.12	FLJ
11	Isoforsythoside	C_29_H_36_O_15_	647.19324 [M+Na]^+^, 321.08435,347.21368	16.12	FF
12	Rutin	C_27_H_30_O_16_	611.16066 [M+H]^+^, 164.97000,229.01273, 256.98975, 285.01004	16.23	FLJ, FF
13	Isoquercitin	C_21_H_20_O_12_	465.10275 [M+H]^+^, 57.06033,164.96709, 229.02371, 285.05786	16.32	FLJ, FF
14	Hyperoside	C_21_H_20_O_12_	465.10275 [M+H]^+^, 257.06033,164.96709, 229.02371, 285.05786	16.65	FLJ, FF
15	Luteoloside	C_21_H_20_O_11_	449.10784 [M+H]^+^, 152.88036	17.08	FLJ
16	Forsythoside B	C_34_H_44_O_19_	779.23690 [M+Na]^+^	18.21	FF
17	Forsythoside A	C_29_H_36_O_15_	647.19464 [M+Na]^+^, 321.08435,347.21368	18.45	FF
18	3, 5-dicaffeoylquinic acid	C_25_H_24_O_12_	517.13405 [M+H]^+^, 162.96284,319.11139	19.15	FLJ
19	Astragalin	C_21_H_20_O_11_	449.10784 [M+H]^+^, 152.88036	19.20	FLJ
20	Genistin	C_21_H_20_O_10_	433.11292 [M+H]^+^	19.99	FLJ
21	Pinoresinol-β-D-glucoside	C_26_H_32_O_11_	543.18368 [M+Na]^+^, 219.06879,291.10626, 142.71555, 113.25488	20.46	FF
22	Epipinoresinol-β-D-glucoside	C_26_H_32_O_11_	543.18231 [M+Na]^+^, 142.98523,219.06894, 231.05322, 266.29376,281.40131, 291.15970	20.96	FF
23	3, 4-dicaffeoylquinic acid	C_25_H_24_O_12_	517.13405 [M+H]^+^, 162.96284,319.11139	21.63	FLJ
24	Pinoresinol monomethyether-β-D-glucoside	C_25_H_30_O_11_	529.16803 [M+Na]^+^	23.31	FF
25	Arctiin	C_27_H_34_O_11_	557.19933 [M+Na]^+^	24.19	FF
26	Quercetin	C_15_H_10_O_7_	303.04993 [M+H]^+^	24.91	FLJ, FF
27	Luteolin	C_15_H_10_O_6_	287.05501 [M+H]^+^	25.20	FLJ
28	Genistein	C_15_H_10_O_5_	271.06010 [M+H]^+^	25.30	FLJ
29	Phillyrin	C_27_H_34_O_11_	557.19933 [M+Na]^+^, 291.15938,249.07809, 218.97397, 143.08472	25.67	FF
30	Macranthoidin B	C_65_H_106_O_32_	1421.65594 [M+Na]^+^	28.82	FLJ
31	Dipsacoside B	C_53_H_86_O_22_	1097.55030 [M+Na]^+^	28.89	FLJ
32	Phillygenin	C_21_H_24_O_6_	373.16456 [M+H]^+^	28.90	FF
33	Pinoresinol	C_20_H_22_O_6_	359.14891 [M+H]^+^, 355.15335,	28.94	FF
34	Epipinoresinol	C_20_H_22_O_6_	359.14847 [M+H]^+^	29.29	FF
35	Arctigenin	C_21_H_24_O_6_	373.16456 [M+H]^+^	29.95	FF
36	Unknown	C_24_H_30_O_6_	437.19244 [M+Na]^+^, 168.99055,259.07562, 243.24869, 285.09967, 200.95114	31.78	FLJ, FF
37	Unknown	C_24_H_41_	330.33511 [M+H]^+^, 101.99937,268.31992, 284.25348, 294.42615	32.64	FLJ, FF
38	Unknown	C_14_H_22_O_2_	223.16858 [M+H]^+^, 139.06036,122.95891, 120.98438, 125.05688	33.24	FLJ, FF
39	Unknown	C_16_H_22_O_4_	279.15869 [M+H]^+^, 101.94276,268.31708, 294.47253	33.91	FLJ, FF
40	Unknown	C_17_H_37_O	280.26303 [M+Na]^+^, 263.10306,245.16504	35.02	FLJ, FF
41	Unknown	C_17_H_35_O	256.26257 [M+H]^+^, 70.93882	35.66	FLJ, FF
42	Unknown	C_27_H_50_O_15_	637.30347 [M+Na]^+^, 525.16754,469.17981	35.80	FLJ, FF
43	Unknown	C_19_H_37_O	282.27856 [M+H]^+^, 247.24356,265.08508	35.86	FLJ, FF
44	Unknown	C_19_H_39_O	284.29425 [M+H]^+^, 87.93765,101.98130, 115.97544	36.79	FLJ, FF
45	Unknown	C_5_O_9_	226.95065 [M+Na]^+^, 158.86320,90.85480	37.13	FLJ, FF
46	Unknown	C_7_H_4_	111.01971 [M+Na]^+^, 98.79458	37.51	FLJ, FF

### The bioavailability interaction of main components from FLJ and FF using *in* *vivo* pharmacokinetics study

**Figure 5:**
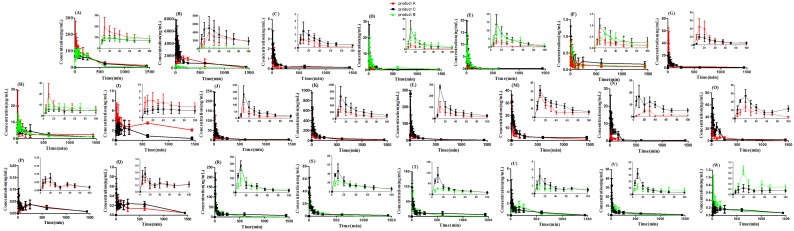
Mean pharmacokinetic profiles of caffeic acid (A), quinic acid (B), luteoloside (C), rutin (D), hyperoside (E), isoquercitrin (F), luteolin (G), quercetin (H), genistein (I), neochlorogenic acid (J), chlorogenic acid (K), cryptochlorogenic acid (L), 3, 5-dicaffeoylquinic acid (M), 3, 4-dicaffeoylquinic acid (N), loganin (O), genistin (P), astragalin (Q), forsythoside A (R), forsythoside B (S), isoforsythoside (T), pillyrin (U), pinoresinol-β-D-glucoside (V) and arctigenin (W) following oral administration of product A, B and C groups.

**Figure 6:**
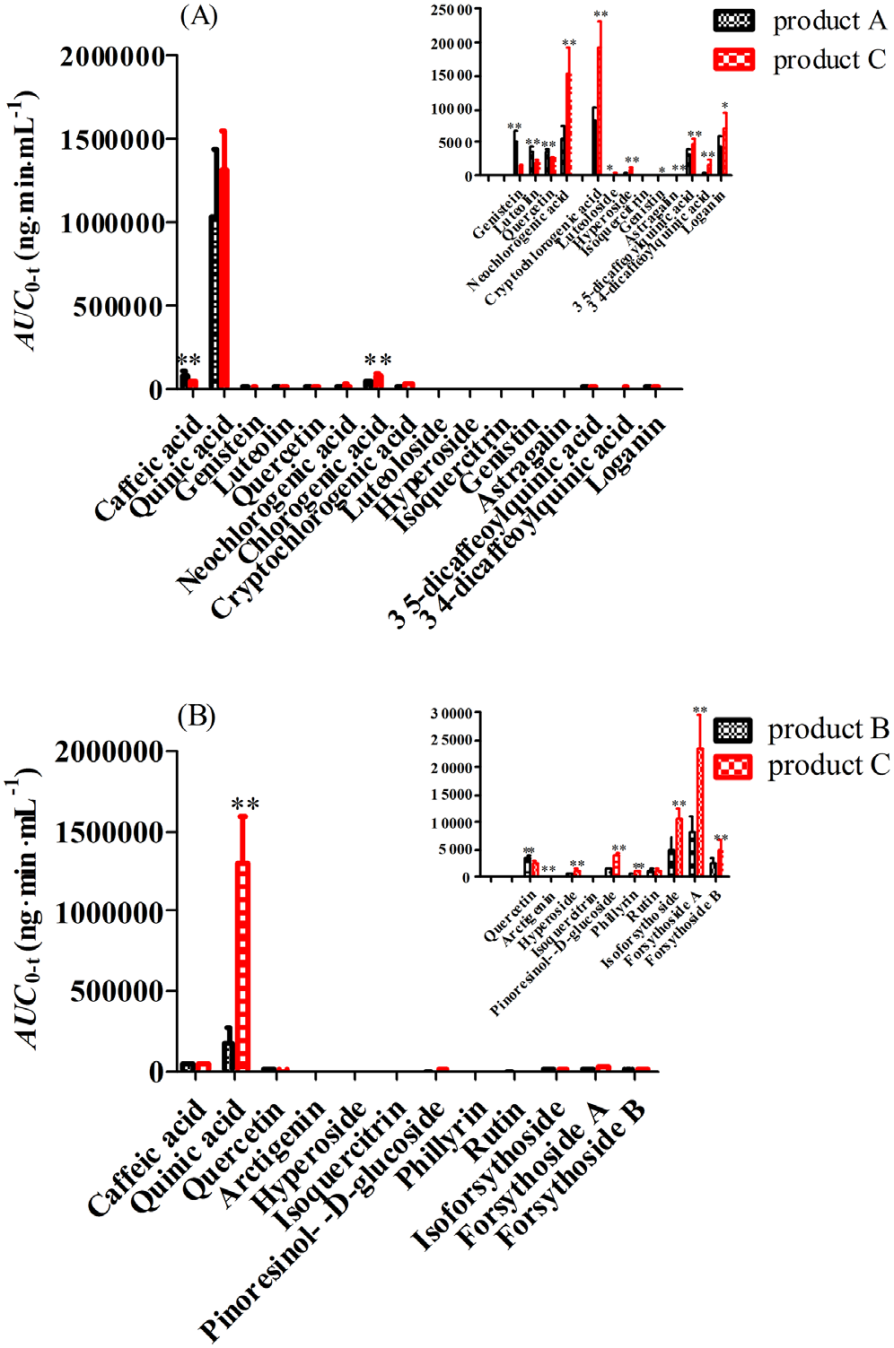
The Pharmacokinetic parameter (*AUC*) of main ingredients as variates analyzed by both HCA and PCA. (*) *P*<0.05 and (**) *P*<0.01, compared with product C group. (A: *AUC* between product A and product C group; B: *AUC* between product B and product C group).

As shown in Fig. 5 and Table 2, *C*_max_, *MRT*, *T*_1/2z_ and *AUC*_0→∞_ of caffeic acid derivatives, flavonoids glucoside, lignins glucoside and iridoids glucoside were improved, but that of flavonoids aglycone and lignins aglycone were decreased in product C group compared with that in product A or B group (most of them had significant differences), which indicated that the intestinal absorption of caffeic acid derivatives, flavonoids glucoside, lignins glucoside and iridoids glucoside might be improved or the transformation from glucoside to aglycone by bacterial metabolism might be inhibited as FLJ combined with FF. In addition, the *AUC*_0→t_ (Fig. 6) as variates analyzed by both HCA and PCA of neochlorogenic acid, chlorogenic acid, cryptochlorogenic acid, 3, 5-dicaffeoylquinic acid, 3, 4-dicaffeoylquinic acid, luteoloside, genistin, astragalin, loganin, pillyrin, pinoresinol-β-D-glucoside, isoforsythoside, forsythoside A and forsythoside B in product C group were increased significantly to 286% (15542±3640.1) ng⋅min/mL, 215% (76148±14584) ng⋅min/mL, 235% (19248±4037.2) ng⋅min/mL, 162% (4944.7±595.54) ng⋅min/mL, 318% (1464.6±309.02) ng⋅min/mL, 137% (285.71±76.804) ng⋅min/mL, 131% (32.880±2.0100) ng⋅min/mL, 145% (250.30±28.344) ng⋅min/mL, 121% (6450.5±484.60) ng⋅min/mL, 213% (3374.0±265.32) ng⋅min/mL, 299% (1025.2±171.44) ng⋅min/mL, 203% (12320±1429.1) ng⋅min/mL, 230% (25229±426.79) ng⋅min/mL and 175% (5642.5±122.33) ng⋅min/mL, respectively, compared to that in product A or B groups, but the *AUC*_0→t_ of genistein, luteolin and arctigenin in product C group were lower significantly when the same dosages were administrated to rats. Besides, the bioavailability of caffeic acid in product C group had no significant difference, compared with that in product B group, and surprisingly significantly lower than that in product A group, and the bioavailability of quinic acid in product C group had no significant difference, compared with that in product A group, and higher significantly than that in product B group although the contents of caffeic acid and quinic acid (Fig. 3) in product C group were significantly higher than that in product A or B group. And the bioavailability of hyperoside in product C group was significantly higher than that in product A or B group, which corresponded to their administration dosages to rats, but that of quercetin was opposite. In addition, the bioavailability of rutin in product C group was significantly higher than that in product A group, but no significant difference compared to that in product B group, and that of isoquercitrin was no significantly different among product A, B and C groups, though the dosage of product C group was higher than that of product A or B group. In short, it was found surprisingly that the bioavailability of some ingredients, such as caffeic acid and quercetin, *etc.* were significantly higher than that in product C, which was contrary to their administration dosages to rats. Therefore, it is necessary for us to consider caffeic acid and quercetin, *etc.* as important variates in the process of PCA and HCA.

**Table 2:** Pharmacokinetic parameters of multi-compounds.

Parameters	*C*_max_ (ng·mL^−1^)			*T*_max_ (min)				*MRT* (min)			*AUC*_0→∞_ (ng·min·mL^−1^)			*T*_1/2z_ (min)		
Products	A	B	C	A	B	C		A	B	C	A	B	C	A	B	C
Caffeic acid	181±17.6**	91.1±6.74	98.1±16.2	10±0.0	10±0.0	10±0.0		399±27.5	334±45.1	377±51.5	71597±5695.4**	40993±10833	44196±2835.1	360±66.3	332±53.6	392±61.1
Quinic acid	383*10±182*10	358±157**	500*10±285*10	21±9.9	18±9.6	10±0.0		239±32.9**	270±18.2**	441±22.6	13808*10^2^±19992*10	17980*10±34769**	14901*10^2^±21646*10	229±16.8**	246±37.7**	330±76.3
Genistein	5.09±2.46*	―	2.30±1.00	14±5.3	―	15±5.8		650±18.6**	―	463±17.1	14425±1481.2	―	1715.2±417.45	600±76.4**	―	411±67.7
Luteolin	44.9±19.8**	―	19.5±5.43	10±0.0	―	10±0.0		347±28.4**	―	280±39.2	5296.2±737.73**	―	2816.7±543.87	423±56.6**	―	265±15.7
Quercetin	15.6±4.39**	12.6±2.23**	8.02±0.493	10±0.0	10±0.0	10±0.0		493±75.0	420±18.2	427±49.7	8667.6±1751.4**	3899.7±193.44	3143.6±592.07	277±52.6	316±55.8	280±62.9
Neochlorogenic acid	75.7±30.1**	―	181±65.0	10±0.0	―	10±0.0		322±33.8**	―	387±62.1	8298.0±1058.7**	―	24226±4891.0	458±38.2**	―	515±28.0
Chlorogenic acid	367±169	―	661±294*	10±0.0	―	10±0.0		347±28.3**	―	497±44.0	42426±5765.9**	―	10366*10±8547.5	322±44.0**	―	461±32.1
Cryptochlorogenic acid	352±29.4**	―	449±22.8	10±0.0	―	10±0.0		333±26.3**	―	453±27.2	11757±1572.1**	―	21078±1913.4	104±46.9**	―	279±17.2
Arctigenin	―	0.895±0.166**	0.231±0.144	―	20±0.0	20±0.0		―	560±32.1	564±26.6	―	266.53±5.8286**	239.53±9.7188	―	623±21.6	534±53.9
Genistin	0.0550±0.0154*	―	0.0753±0.0285	16±8.7	―	20±0.0		650±18.6**	―	463±17.1	37.8±1.94*	―	49.3±3.02	482±18.3*	―	552±27.3
Luteoloside	1.26±0.655	―	2.48±1.34	10±0.0	―	10±0.0		347±28.4**	―	280±39.2	177.91±30.147**	―	300.16±32.890	383±13.2**	―	579±85.7
Astragalin	0.441±0.166	―	0.551±0.207	10±0.0	―	10±0.0		489±29.3*	―	680±109	259.05±30.440**	―	375.45±42.516	460±15.8*	―	695±103
Hyperoside	5.25±0.859**	9.68±4.39**	22.1±1.90	10±0.0	―	10±0.0		436±24.4	409±60.9	387±41.8	593.59±19.662**	625.17±111.23**	1473.1±161.12	410±97.9	401±29.2	423±41.3
Isoquercitrin	0.504±0.351**	0.619±0.212**	1.00±0.300	10±0.0	20±0.0	16±8.7		557±46.9	498±66.7	555±46.9	158.09±27.790**	195.20±29.918**	330.89±56.587	436±26.3	471±30.9	455±52.1
3, 5-dicaffeoylquinic acid	27.6±4.73	―	30.8±5.66	10±0.0	―	10±0.0		438±25.9**	―	578±18.1	4291.1±243.43**	―	5384.9±198.59	292±77.6**	―	440±47.4
3, 4-dicaffeoylquinic acid	4.79±1.87**	―	12.5±4.07	15±4.6	―	12±4.1		422±13.5**	―	537±34.1	1376.6±251.42**	―	2327.6±152.15	295±37.0**	―	409±142.6
Rutin	4.08±1.99**	14.6±1.65	19.8±3.93	10±0.0	―	10±0.0		301±50.9	261±29.2	293±51.6	618.79±104.54**	1828.0±149.21	1366.6±329.59	476±50.2	490±92.9	453±36.8
Loganin	28.0±4.93**	―	54.7±8.63	21±3.8	―	16±8.1		349±36.4**	―	470±57.0	5571.8±490.50**	―	8381.5±1477.5	124±12.3**	―	440±16.4
Pinoresinol-β-D-glucoside	―	11.9±4.36**	32.3±7.57	―	10±0.0	10±0.0		―	246±33.4**	454±30.3	―	1917.7±480.53**	4067.3±345.77	―	202±12.6**	315±28.3
Phillyrin	―	2.24±0.978**	4.41±0.0769	―	10±0.0	10±0.0		―	316±11.8**	521±55.3	―	389.31±94.693**	1316.5±171.15	―	341±19.2**	450±52.4
Isoforsythoside	―	71.7±13.5**	188±33.7	―	10±0.0	10±0.0		―	240±15.5**	452±36.6	―	6736.9±861.30**	12347±1731.7	―	202±36.0**	455±38.5
Forsythoside A	―	102±6.19*	182±38.6	―	10±0.0	10±0.0		―	225±38.7**	452±27.0	―	13728±4154.2	46409±6015.3**	―	276±62.4**	359±21.4
Forsythoside B	―	25.5±9.37*	46.6±4.91	―	10±0.0	10±0.0		―	198±26.7**	380±43.3	―	3249.3±268.96*	5223.2±666.32	―	259±36.4**	425±50.7

(*) *p*<0.05 and (**) *p* <0.01 compared with product C.

### Pattern recognition analysis of pharmacokinetic profiling *in* *vivo*

**Figure 7:**
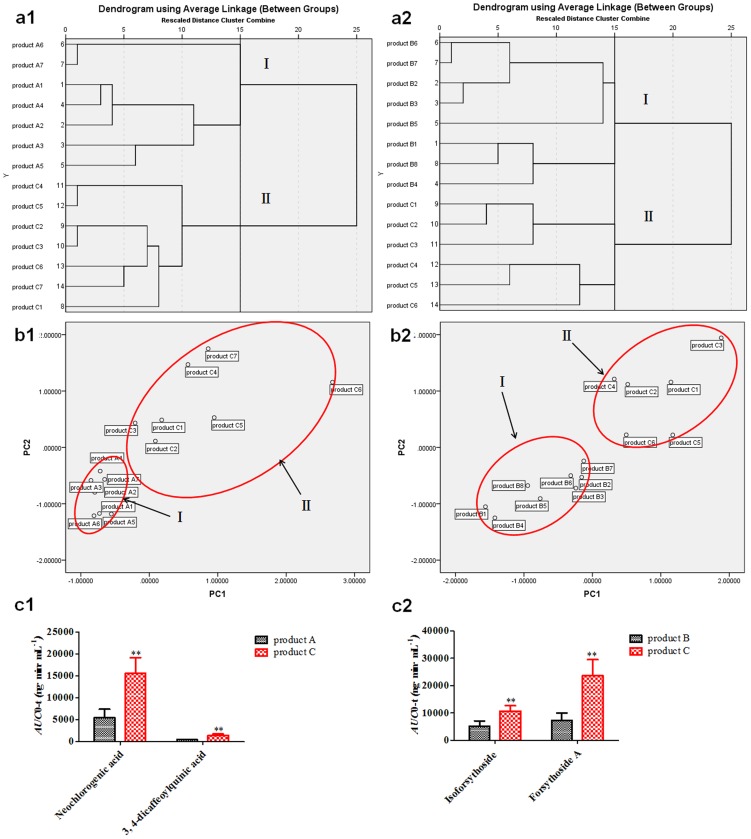
The pharmacokinetics differences among product A, B and C groups analyzed by both HCA and PCA. (a1, a2: HCA; b1, b2: PCA; The most important ingredients influencing the difference between product C and product A group (c1) or between product C and product B group (c2)).

Hierarchical clustering of the pharmacokinetics was presented in Fig. 7a1 and a2. The dendrogram showed the cluster relationships among product A, product B and product C groups *in vivo*. We found that the samples were divided into two main clusters, respectively. As shown in Fig. 7a1, cluster I represented product A1–A7, and cluster II grouped product C1–C7, which was illustrated by PCA (Fig. 7b1) by which samples were also clearly separated two domains, consistent with HCA analysis. Also in Fig. 7a2, product B1–B7 was marked in cluster I, and product C1–C6 was divided into cluster II, which was proved by PCA (Fig. 7b2) that samples were clearly grouped into two domains, correspondence to HCA analysis. In addition, it was displayed (Fig. 7c1) that the exposure level *in vivo* of one group of ingredients (neochlorogenic acid and 3, 4-dicaffeoylquinic acid) were the main influencing factor contributing to the difference between product C and product A group by both PCA and HCA. Similar results were obtained in the case of another group of ingredients (isoforsythoside and forsythoside A) between product C and product B group (Fig. 7c2).

### The absorption interaction of main component from FLJ and FF using *in* *vitro* Caco-2 cells model

Caco-2 cells were exposed to various concentrations of product A, B and C (0.039, 0.078, 0.16, 0.31, 0.63, 1.3, 2.5, 5.0, 10 and 20 mg raw medicine per milliliter. It was shown that product A, B and C at different concentrations were all safe for the Caco-2 cells from MTT test.

**Figure 8:**
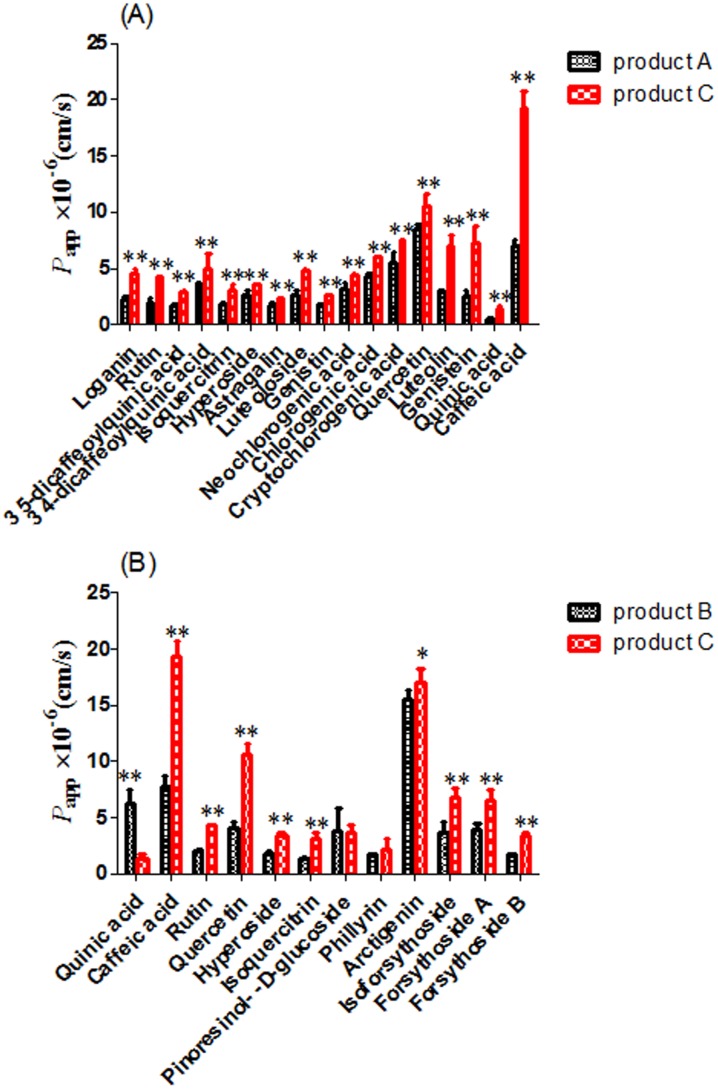
The intestinal absorption *in vitro* (*P*_app_) of main ingredients as variates analyzed by both HCA and PCA. (*) *P*<0.05 and (**) *P*<0.01, compared with product C group. (A: *P*_app_ between product A and product C group; B: *P*_app_ between product B and product C group).

As shown in Fig. 8A, the *P*_app_ for caffeic acid, genistein, luteolin, quercetin, neochlorogenic acid, chlorogenic acid, cryptochlorogenic acid, 3, 5-dicaffeoylquinic acid, 3, 4-dicaffeoylquinic acid, genistin, luteoloside, astragalin, hyperoside, isoquercitrin, rutin and loganin in product C group were increased significantly to 275%, 293%, 240%, 125%, 136%, 140%, 133%, 164%, 137%, 165%, 174%, 131%, 129%, 169%, 213% and 211% respectively compared to that in product A group, and the *P*_app_ (Fig. 8B) was increased to 247%, 256%, 195%, 234%, 210%, 110%, 187%, 165% and 210% for caffeic acid, quercetin, hyperoside, isoquercitrin, rutin, arctigenin, isoforsythoside, forsythoside A and forsythoside B, respectively compared with that in product B group, though some of them had different concentrations. Besides, the *P*_app_ for quinic acid in product C group was increased to 301% compared to that in product A group, but decreased to 24.0% compared to that in product B group. Meanwhile, there were no significant difference in *P*_app_ for pillyrin and pinoresinol-β-D-glucoside between product C group and B group. In addition, we found that dipsacoside B and macranthoidin B in product C or A group and arctiin in product C or B group might not be absorbed well into Caco-2 cells. The result above showed that the *P*_app_ for most of ingredients such as caffeic acid derivatives were improved significantly by FLJ combined with FF, which might be attributed to the influence of components in FLJ or FF on the efflux transporters.

### Pattern recognition analysis of absorption profiling *in* *vitro*

**Figure 9:**
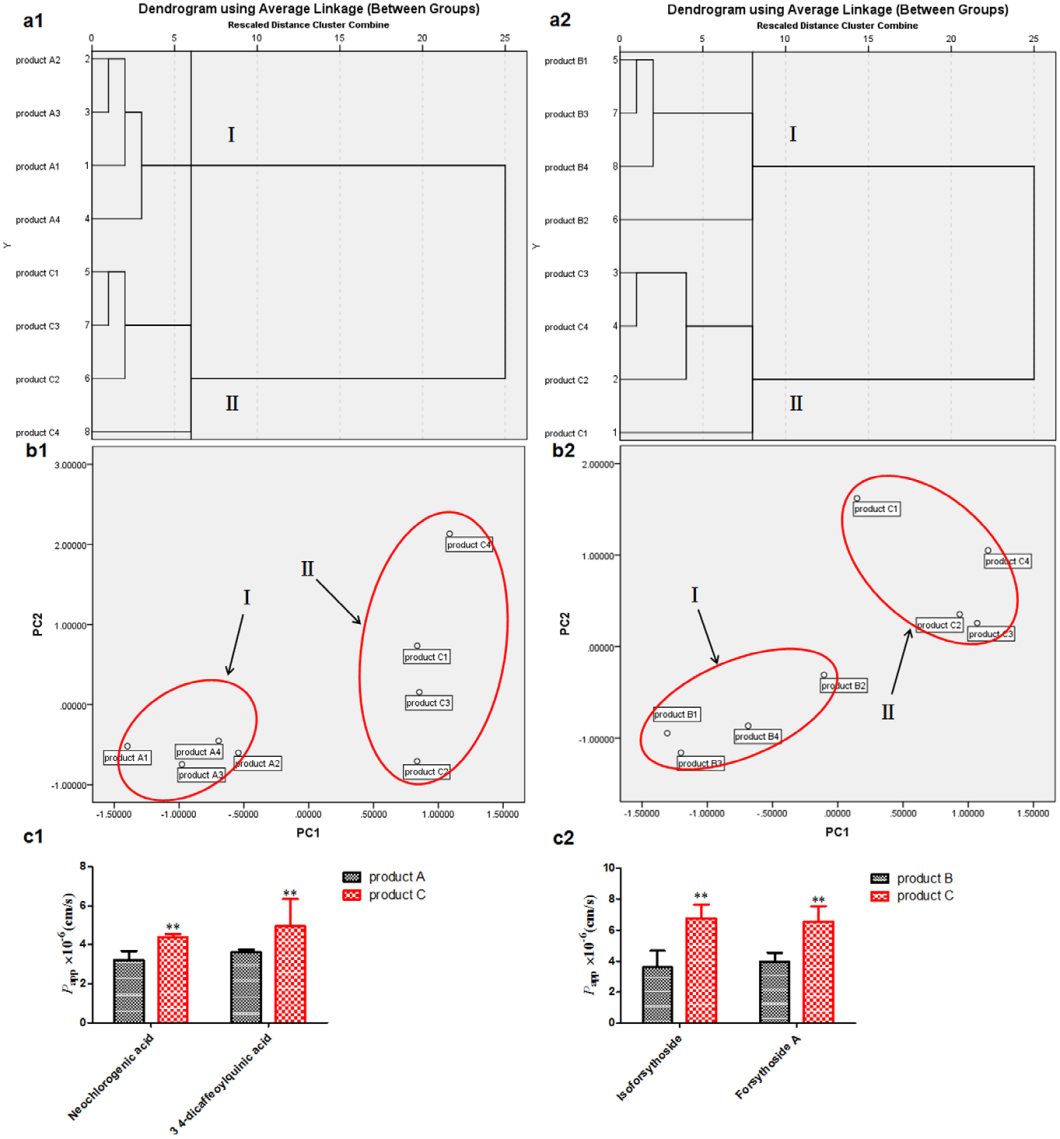
The absorption differences among product A, B and C groups analyzed by both HCA and PCA. (a1, a2: HCA; b1, b2: PCA; The most important ingredients influencing the difference between product C and product A group (c1) or between product C and product B group (c2)).

Fig. 9a1 and a2 showed the hierarchical clustering of the absorption *in vitro*, and we found the samples were divided into two main clusters, respectively. As shown in Fig. 9a1, cluster I represented product A1–A4, and cluster II grouped product C1–C4, which was illustrated by PCA (Fig. 9b1) that samples were also clearly separated two domains, consistent with HCA analysis. Besides, it was shown (Fig. 9a2) that product B1–B4 was marked in cluster I, and product C1–C4 was divided into cluster II, which was also proved by PCA (Fig. 9b2) that samples were clearly grouped into two domains, correspondence to HCA analysis. Meanwhile, it was displayed (Fig. 9c1) that the intestinal absorption level *in vitro* for one group of ingredients (neochlorogenic acid and 3, 4-dicaffeoylquinic acid) were the main influencing factors leading to the difference between product C and product A group. Similar results were obtained in the case of another group of ingredients (isoforsythoside and forsythoside A) between product C and product B group (Fig. 9c2).

## Discussion

In the present study, the systemic biopharmaceutics combined with HCA and PCA were firstly performed to rapidly elucidate the compatibility foundation of FLJ-FF herb couple, and it was found consistently (Fig. 4 & 7) that the caffeic acid derivatives including isoforsythoside, 3, 5-dicaffeoylquinic acid, neochlorogenic acid, 3, 4-dicaffeoylquinic acid and forsythoside A were the most important ingredients resulting in difference between herb couple and single herbs, and interestingly, their exposure levels in the views of not only chemistry *in vitro* but also biology *in vivo* were all significantly higher than that in single herbs, highly consistent with the data that the pharmacological activities such as anti-inflammatory and antipyretic effects in FLJ-FF herb couple were significantly higher than that in FLJ or FF [17]. Besides, it was reported that caffeic acid derivatives such as isochlorogenic acids and forsythoside A had strong antioxidant, antibacterial and antiviral activities [18]–[20], and chito-oligosaccharide (COS) at dosage of 25 mg/kg could improve their pharmacological effects such as antiviral activity via enhancing the intestinal permeabilities and the *in vivo* bioavailabilities of caffeic acid derivatives significantly in FF-FLJ herb couple preparations [11]. The studies above indicated that the caffeic acid derivatives might be the most significant components contributing to the pharmacological effects.

It was shown (Fig. 9) from Caco-2 cells *in vitro* combined with HCA and PCA that neochlorogenic acid, 3, 4-dicaffeoylquinic acid, isoforsythoside and forsythoside A were the main ingredients resulting in the difference between single herbs and herb couple, and the values of intestinal absorption *in vitro* were significantly higher than that in single herbs, surprisingly consistent with the results obtained from pharmacokinetics *in vivo* (Fig. 7), which indicated that it was because of intestinal absorption improvement of neochlorogenic acid, 3, 4-dicaffeoylquinic acid, isoforsythoside and forsythoside A that the efficacies of herb couple were better than that of single herbs.

In the previous study, we found that the poor intestinal absorption of the caffeic acid derivatives was one of the most important factors resulting in the low oral bioavailability, and they permeated mainly via the paracellular pathways in the intestine, and the intestinal absorption of phenolic acid such as chlorogenic acid, neochlorogenic acid, cryptochlorogenic acid, 3, 4-dicaffeoylquinic acid and 3, 5-dicaffeoylquinic acid were influenced by P-gp, MRP2 and BCRP, but that of phenylethanoid glycosides such as forsythoside A, isoforsythoside and forsythoside B were affected by P-gp and MRP2, not BCRP [21]–[22]. Although the bioavailability of active ingredients (Fig. 6) (neochlorogenic acid, 3, 4-dicaffeoylquinic acid, isoforsythoside and forsythoside A) in herb couple were improved compared with that in single herbs, their intestinal absorptions (Fig. 8) were still unsatisfactory. Thus, the studies on how to improve the bioavailability of the caffeic acid derivatives in herb couple by pharmaceutical methods such as absorption enhancers based on tight junctions need to be further investigated.

## Conclusion

Current findings from both the chemical and biological aspects consistently demonstrated that the biopharmaceutics characteristics (dissolution *in vitro* and intestinal absorptions both *in vivo* and *in vitro*) of caffeic acid derivatives in FLJ-FF herb couple, higher than that in FLJ or FF, contributed to the optimal efficacy of herb couples analyzed by both HCA and PCA, which indicated that caffeic acid derivatives should be considered as chemical markers to control the quality of its preparations.
